# Testing the Potential of Regulatory Sigma Factor Mutants for Wastewater Purification or Bioreactor Run in High Light

**DOI:** 10.1007/s00284-020-01973-w

**Published:** 2020-04-07

**Authors:** Dimitar Valev, Juha Kurkela, Esa Tyystjärvi, Taina Tyystjärvi

**Affiliations:** Department of Biochemistry/Molecular Plant Biology, University of Turku, 20014 Turku, Finland

## Abstract

It is shown that a freshly inoculated culture of the model cyanobacterium *Synechocystis* sp. PCC 6803 consumed almost all phosphate and 50% of nitrate within 6 days from the nutrient-rich BG-11 growth medium, indicating potential of cyanobacteria to purify wastewaters. *Synechocystis* sp. PCC 6803 control strain also collected nutrients efficiently from a landfill leachate wastewater KA2 (5.9–6.9 mM ammonium and 0.073–0.077 mM phosphate). Wastewaters might induce oxidative stress to microalgae, which prompted us to test growth of sigma factor inactivation strains, as ΔsigBCE and ΔsigCDE strains show superior growth in chemically induced oxidative stress. All cyanobacterial strains, including a stress-sensitive strain ΔsigBCDE, grew well in KA2 for four days, indicating that KA2 did not cause immediate oxidative stress. Completely arrested growth and bleaching of ΔsigBCDE cells after one week in KA2 wastewater point to the importance of group 2 sigma factor-mediated changes in gene expression during wastewater treatment. The growth of ΔsigBCD was arrested early in un-buffered and Hepes buffered (pH 7.5) KA2. In ΔsigBCD, all phosphate transporter genes are upregulated in standard conditions, and ΔsigBCD cells showed growth defects in low-phosphate BG-11 medium. ΔsigBCD cells removed phosphate slower from KA2 than the control strain, but phosphate supplementation of KA2 did not improve growth of ΔsigBCD. The ΔsigBCE strain showed superior growth in a laboratory-scale bioreactor in bright light and removed phosphate even slightly more efficiently than the control strain if KA2 was Hepes buffered although ΔsigBCE grew slowly in un-buffered KA2 and in low-phosphate BG-11 medium. The results indicate that engineering expression of regulatory group 2 sigma factor(s) might be useful for practical applications.

**Electronic supplementary material:**

The online version of this article (10.1007/s00284-020-01973-w) contains supplementary material, which is available to authorized users.

## Introduction

Concerns about the environment have promoted research aiming at finding alternatives for efficient recycling of resources and production of food and fuel in a carbon neutral way. Municipal, piggery and dairy wastewaters, and anaerobic digestion reject waters are just few examples of nutrient-rich wastewaters, containing plenty of ammonium, nitrate, and phosphate [1, 2]. Such wastewaters cause eutrophication of natural waters. Microalgal-based wastewater treatment might offer an alternative or supplement to the conventional wastewater treatment processes. In the simplest approach for wastewater purification with microalgae, the algae absorb inorganic nutrients and utilize those for biomass production, for a review see [3]. Biomass, in turn, can be used for energy production or as a fertilizer. In a more ambitious scenario, cyanobacteria could be engineered to produce high-value compounds, not only biomass, and simultaneously remediate wastewaters.

Cyanobacteria can utilize nitrate [4], ammonium [5], and urea [6] as their nitrogen source. Ammonium ion enters the *Synechocystis* sp. PCC 6803 cell via permeases Amt1, 2, and 3 [7]. The nitrite--nitrate transporter NTR takes in nitrate and then nitrate reductase NarB reduces it to nitrite and nitrite reductase NirA further on to ammonium, for a recent review see [8]. Although cyanobacteria prefer ammonium, high concentrations of ammonium are toxic. *Synechocystis* sp. PCC 6803 grows similarly in high CO_2_ whether 17.6 mM ammonium or 17.6 mM NaNO_3_ is used as a nitrogen source [9], but in ambient air, ammonium concentrations above 10 mM adversely affect PSII activity [10]. If excess nitrogen is available, then cyanobacteria balance metabolism by producing the storage compound cyanophycin [11].

The other main nutrient, phosphorus, is taken up by two phosphate-specific transporters, Pst1 and Pst2 [12]. The main transporter, Pst1, is induced during phosphate limitation [13]. When excess of inorganic phosphate is available, cyanobacteria store phosphate as polyphosphate and then utilize it during phosphate starvation using two inducible enzymes exopolyphosphatase Ppx and inorganic pyrophosphorylase Ppa [14]. Phosphate metabolism is regulated by a two-component system consisting of a histidine kinase SphS and a response regulator SphR and by a negative regulator SphU [15–17]. To achieve fast and stable growth, aquatic phototrophs require adequate concentrations of N and P and in some cases P or N have to be adjusted for long-term cultivations in wastewaters [18, 19].

For biotechnical applications, robust strains with high acclimation capacity to suboptimal conditions are beneficial, as keeping up constant optimal environmental conditions is expensive. Acclimation of cyanobacteria to suboptimal conditions depends strongly on changes in gene expression. In cyanobacteria, the regulatory σ subunits of the RNA polymerase (RNAP) play major roles in acclimation responses [20]. In optimal growth conditions, the RNAP core mainly recruits the primary σ factor that guides RNAP to transcribe housekeeping genes, while in suboptimal conditions, alternative σ factors are recruited more regularly, and the expression of genes necessary for acclimation to that particular stress is upregulated [21]. Biotechnical engineering of σ factors might be useful to produce more robust expression strains and offer a way to control production of valuable compounds [22]

The model cyanobacterium *Synechocystis* sp. PCC contains nine σ factors. SigA is an essential primary σ factor; SigB, SigC, SigD, and SigE are group 2 σ factors; and SigF, SigG, SigH, and SigI are group 3 σ factors [20]. Recent studies indicate importance of group 2 σ factors in acclimation processes. *Synechocystis* s. PCC 6803 cells grow well without group 2 σ factors in optimal conditions, but the group 2 σ factor-deficient ΔsigBCDE cells have lost their capacity to acclimate to stress conditions [21] (see Table 1). The current knowledge about the phenotypes of the triple inactivation strains containing only one functional group 2 σ factor have been collected to Table 1. The SigB factor plays a crucial role when cells acclimate to heat [23, 24] or high salt [25, 26] stresses. Furthermore, a high SigB content protects cells against photoinhibition [27, 28] and toxicity of butanol [29] and offers protection against hydrogen peroxide stress and high temperature [29, 30]. Accumulation of RNAP-SigC holoenzyme keeps cyanobacterial cells in the stationary phase [31, 32], while the SigE factor activates sugar catabolic reactions in darkness [21, 33, 34]. The SigD factor plays a major role in acclimation to bright light and singlet oxygen stresses [20, 30, 35, 36].

**Table 1:** Properties of group 2 σ factor triple and quadruple inactivation strains of *Synechocystis* sp. PCC 6803

Strain	Group 2 σ factor content	Phenotype	References
ΔsigBCD	SigE, 1.5 × that in CS	Sensitive to superoxide	[30]
		Salt sensitive	[26]
		Low temperature sensitive	[37]
		Sensitive to PSII damage	[37]
		Slow growth in mixotrophic conditions	[37]
ΔsigBCE	SigD, 2 × that in CS	Resistant to singlet oxygen stress	[30]
		Resistant to H_2_O_2_ stress	[30]
		Fast growth in moderate and bright light	[26, 30]
		Sensitive to PSII damage	[37]
		Slow growth in blue light	[37]
		Locked in state 1	[37]
ΔsigBDE	Normal amount of SigC	Sensitive to superoxide	[30]
		Low temperature sensitive	[37]
		Sensitive to PSII damage	[37]
		Slow recovery from nitrogen deficiency	[32]
		Salt sensitive	[26]
ΔsigCDE	SigB, 2 × that in CS	Resistant to H_2_O_2_ stress	[30]
		Salt tolerant	[25, 26]
		Photoinhibition tolerant	[27, 28]
		High carotenoid content	[27]
ΔsigBCDE	None	Sensitive to any oxidative stress	[30]
		Salt sensitive	[21]
		Bright light sensitive	[21]
		Heat sensitive	[21]
		Sensitive to nitrogen deficiency	[32]

When three out of four group 2 σ factors are inactivated, the amount of the remaining group 2 σ factor is either doubled (if SigB or SigD is the remaining σ factor), slightly enhanced (SigE), or remains similar (SigC) as in the control strain containing all σ factors [30], see Table 1. As upregulation of either SigB or SigD offers extra protection against oxidative stress [30], it was tested if specific properties offered by group 2 σ factor inactivation strains would be beneficial for practical applications.

## Materials and Methods

### Strains and Standard Growth Conditions

The glucose-tolerant strain of *Synechocystis* sp. PCC 6803 was used as a control strain (CS) in these experiments and as a host strain for the construction of σ factor inactivation strains [37]. Construction of the σ factor inactivation strains ΔsigBCD, ΔsigBCE, ΔsigBDE, ΔsigCDE [37], and ΔsigBCDE [21] has been described earlier. If not otherwise stated, cells were grown in BG-11 medium supplemented with 20 mM Hepes–NaOH, pH 7.5, under constant illumination, photosynthetic photon flux density (PPFD) of 40 µmol m^−2^ s^−1^, at 32 °C in ambient air. For plates, agar (Bacto) was added 15 g/l, and plates for triple and quadruple mutants were supplemented with chloramphenicol (10 µg/ml), kanamycin (20 µg/ml), spectinomycin (10 µg/ml), and streptomycin (20 µg/ml), and for quadruple mutant, also nourseothricin (10 µg/ml) was added. For growth experiments, 30 ml cultures were grown in 100-ml Erlenmeyer flasks without antibiotics, in constant shaking at 90 rpm. Typically, OD_730_ was set to 0.1 (Genesys 10S UV–VIS, Thermo Fisher Scientific, US) at the beginning of each experiment and growth was followed by measuring OD_730_. Before measurements, dense cultures were diluted so that OD_730_ did not exceed 0.4 and the dilutions were taken into account when the results were calculated.

Low-phosphate experiments were done by inoculating cells grown on BG-11 agar plates into BG-11-5%P medium that contains only 8.75 µM K_2_HPO_4,_ 5% of that in the standard BG-11 medium.

### Consumption of Nitrate, Ammonium, and Phosphate

To estimate ammonium, nitrate, or phosphate consumption, aliquots for measurements were taken every 24 h. Cells were removed by filtering the culture trough 0.2 µm cellulose acetate membrane filter (Whatman), and NH_4_^+^, NO_3_^−^, and PO_4_^3−^ were measured from the supernatant with Spectroquant® Ammonium, Nitrate, and Phosphate tests (Millipore) according to the manufacturer’s instructions, respectively.

### KA2 Wastewater

KA2 is contaminated underground water with anaerobic landfill leachate provided by Helsinki Region Environmental Services Authority HSY. Depending on the KA2 batch, it contained ammonium 5.9 to 6.9 mmol/l and phosphate 0.073 to 0.077 mmol/l. The KA2 wastewater had a biochemical oxygen demand/chemical oxygen demand ratio of 0.055 (see Valev et al. [19]), which is the characteristic of “old” landfill leachate [38]. The high turbidity appearance of the KA2 wastewater indicated high total suspended solids that were removed by filtrating through a home-made granular activated carbon filter. Finally, wastewater was sterilized with 0.2 µm PES membrane bottle-top filter (VWR, USA) prior to the experiment. For some experiments, KA2 was supplemented with phosphate to reach the same level as in the BG-11 medium, and for some experiments, the KA2 was also buffered with 20 mM Hepes, pH 7.5, as indicated.

### High-Light Stress

High-light stress conditions were applied in Multicultivator MC1000 device (PSI, Czech Republic). The device consist of eight experimental tubes, submersed in a water bath that maintains the temperature at 32 °C. Cultures were mixed by bubbling with air (circa 60 ml/min), provided by MC1000′s factory supplied air pump. Light conditions for each tube were adjusted to the PPFD of 500 µmol m^−2^ s^−1^ with a submersible light sensor (Model: US-SQS/L, Heinz Walz, Germany). An OD sensor at 730 nm inside each tube was used to monitor growth every 30 min.

## Results

### *Synechocystis* sp. PCC 6803 Cells are Efficient in Acquisition of Nutrients from Growth Medium

**Fig. 1:**
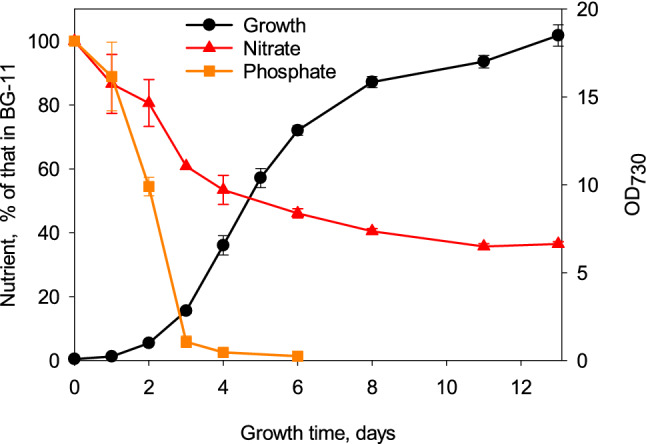
Growth and uptake of nitrate and phosphate from BG-11 growth medium. The growth of *Synechocystis* culture was monitored once a day by measuring OD_730_, and the amounts of remaining nitrate and phosphate in BG-11 growth medium were also measured daily. The nitrate concentration of BG-11 is 17.6 mM and phosphate concentration is 0.175 mM. The pH of the BG-11 growth medium was buffered by adding 20 mM Hepes–NaOH, pH 7.5, and the control strain cells were grown under constant illumination, PPFD 40 µmol m^−2^ s^−1^, at 32 °C. The growth chamber air was enriched with 3% CO_2_. Three independent biological replicates were measured, and the error bars denote SE

To get an idea on how rapidly the cyanobacterium *Synechocystis* sp. PCC 6803 cells acquire nutrients from the growth medium, the glucose-tolerant control strain was grown in BG-11 medium under constant illumination (PPFD 40 µmol m^−2^ s^−1^), at 32 °C in CO_2_-enriched atmosphere, and consumption of nitrate and phosphate was followed for a few days. BG-11 is a rich medium containing 17.6 mM nitrate and 175 µM phosphate. Phosphate was rapidly consumed and only 2% of phosphate remained in the growth medium after four days (Fig. 1), while ~ 50% of nitrate had been consumed at that point. However, rapid growth of cells continued for a week, indicating that acquisition of phosphorus exceeds that needed for growth, and extra phosphorus was stored for future utilization (Fig. 1). Cells continued growth for the whole 13 days of this experiment, but the growth was slow after the first 8 days.

### Landfill Leachate Wastewater is Usable as a Batch Growth Medium for *Synechocystis* sp. PCC 6803

**Fig. 2:**
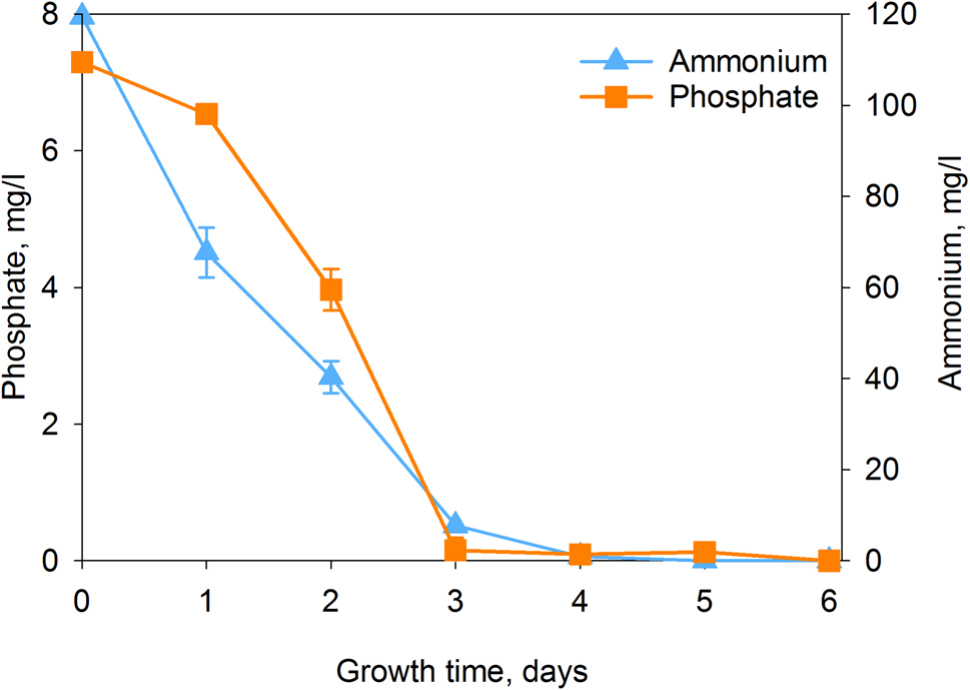
Uptake of ammonium and phosphate from KA2 wastewater. The control strain of *Synechocystis* was grown in KA2 wastewater under constant illumination, PPFD 40 µmol m^−2^ s^−1^, at 32 °C in ambient air. The original ammonium concentration of KA2 wastewater was 6.9 mM (119 mg/ml) and phosphate was 0.077 mM (7.3 mg/l), whereas the nitrate concentration was negligible

**Fig. 3:**
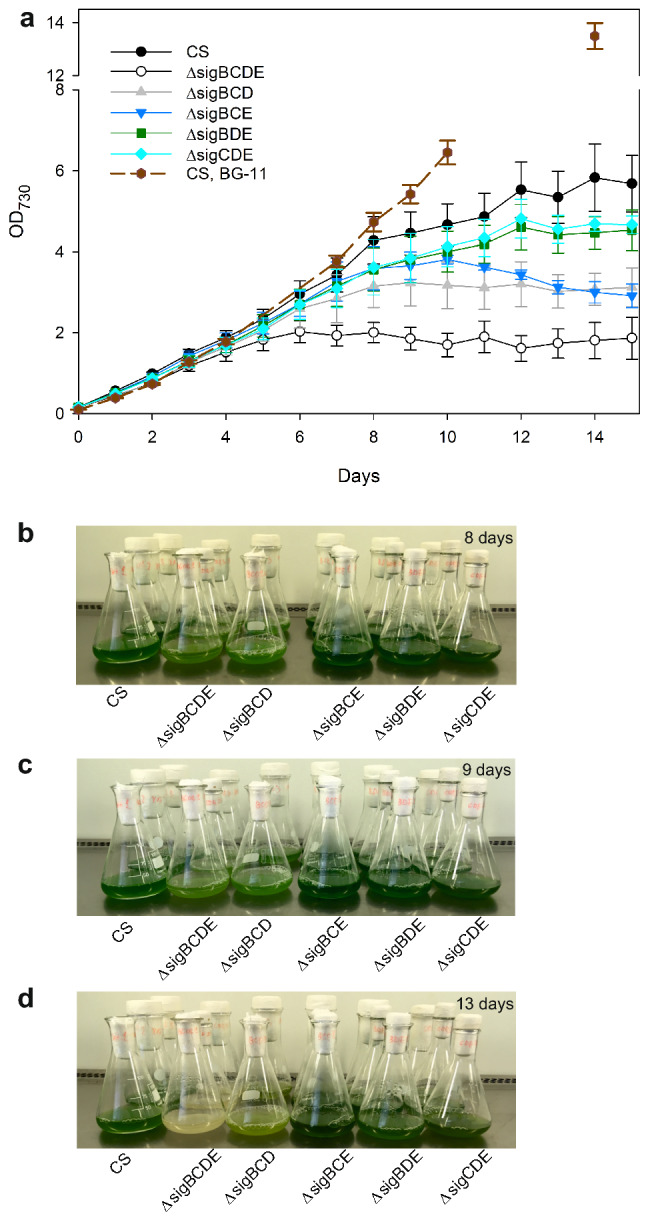
Growth of the control and σ factor mutant strains in wastewater. **a** OD_730_ was set to 0.1 and the growth was followed for 15 days by measuring OD_730_ once a day. For a control experiment, control strain cells were grown in standard BG-11 growth medium. The cells were grown under constant illumination, PPFD 40 µmol m^−2^ s^−1^, at 32 °C in ambient air. Three independent biological replicates were measured, and the error bars denote SE. The wastewater cultures were photographed after 8 (**b**), 9 (**c**), and 13 (**d**) days

As *Synechocystis* cells removed nutrients efficiently from the BG-11 medium, it was next tested how well *Synechocystis* sp. PCC 6803 cells removed nutrients from landfill leachate wastewater KA2 in these standard growth conditions. The KA2 wastewater is poor in nitrate but rich in ammonium (5.9–6.9 mM depending on the batch), and, therefore, the amount of available nitrogen in KA2 is 34–39% of that in BG-11 medium. The phosphate concentration of KA2 was 41–44% of that in BG-11 medium. When the control strain cells were grown under constant illumination (PPFD 40 µmol m^−2^ s^−1^), at 32 °C in KA2 medium in ambient air, only traces of ammonium or phosphate remained in KA after three days (Fig. 2), but cells continued to grow longer, indicating that cyanobacteria store nutrients also from wastewater (Fig. 3).

In addition to the control strain, the growth of the collection of group 2 σ factor mutant strains in KA2 was also tested (Fig. 3). These strains are of interest because ΔsigCDE and ΔsigBCE strains grow better than the control strain in some stress conditions like chemically induced oxidative stress, whereas the ΔsigBCDE strain is vulnerable to all stress conditions [21, 30]. All σ factor triple inactivation strains grow like the control strain in BG-11 medium for 10 days in standard growth conditions [37] and the quadruple mutant ΔsigBCDE almost as well (Supplemental Fig S1). For a comparison of KA2 and BG-11, the control strain was also grown in BG-11 medium.

All strains grew similarly in wastewater as in the BG-11 growth medium for four days (Fig. 3a). The finding that the extremely stress-sensitive ΔsigBCDE strain [21, 32, 39] grew like the control strain for a few days indicates that the KA2 wastewater is devoid of compounds that are immediately toxic to *Synechocystis* sp. PCC 6803 or induce substantial oxidative stress. However, although KA2 wastewater permitted normal growth for a few days, cells grew faster in BG-11 medium than those in KA2 after the first 6 days and reached a higher cell density (Fig. 3a). This suggests that long-term growth in KA2 is a stress for *Synechocystis* sp. PCC 6803. After 5 days, the ΔsigBCDE strain lost the ability to grow (Fig. 3a) and bleaching of pigments became visible after 8 days (Fig. 3b–d). The pale yellow-orange color of the old ΔsigBCDE culture indicated that almost all blue phycobilin pigments and the yellow-green chlorophyll *a* pigments had been degraded, and only some carotenoids remained (Fig. 3d), indicating that specific changes in gene expression are required for long-term growth of *Synechocystis* sp. PCC 6803 in KA2 medium, and this acclimation does not function without group 2 σ factors.

In KA2 wastewater, none of the mutant strains was superior compared to the control strain. ΔsigBDE (contains only SigC) and ΔsigCDE (contains only SigB) continued growth for 11 days just like the control strain, and after the 12th day, the cell content of these strains remained constant and no bleaching was observed (Fig. 3). The cessation of growth occurred already after 8 days for strains ΔsigBCD and ΔsigBCE. The ΔsigBCE strain did not show any bleaching, whereas ΔsigBCD cells turned yellowish-green, indicating bleaching of blue phycobilin pigments in the stationary phase (Fig. 3d).

**Fig. 4:**
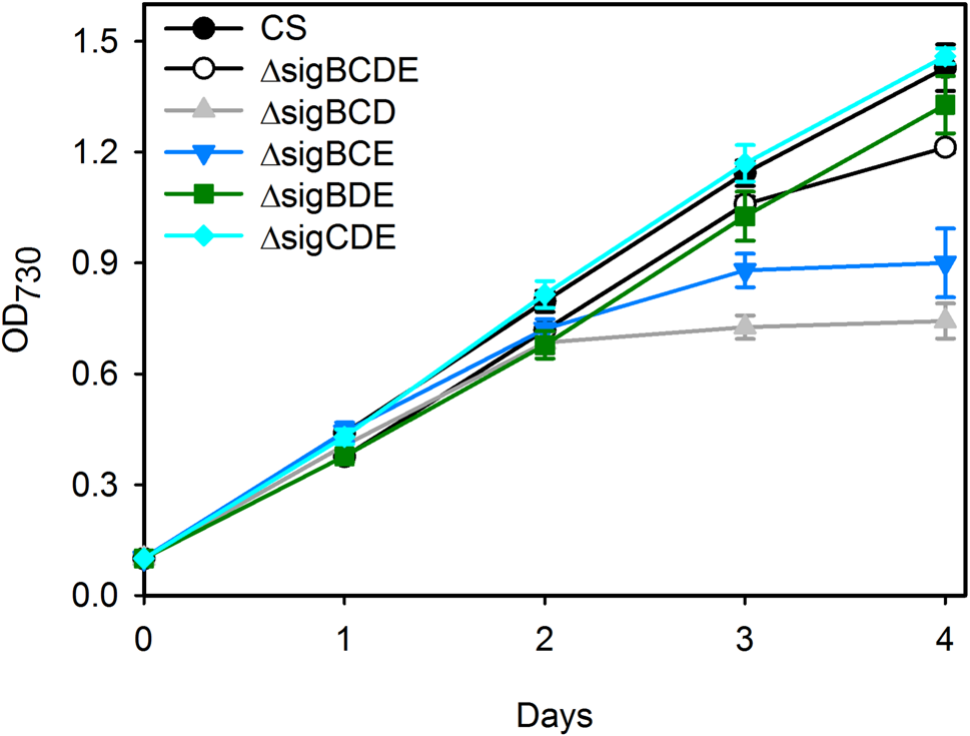
Growth of the control and mutant cells in low-phosphate conditions. The OD_730_ was set to 0.1, and cell cultures were grown in BG-11 medium containing only 5% of the normal phosphate concentration of BG-11 (8.75 µM). The growth medium was buffered with 20 mM Hepes–NaOH, pH 7.5. Cells were grown under constant illumination, PPFD 40 µmol m^−2^ s^−1^, at 32 °C in ambient air. Three independent biological replicates were measured for each strain. The error bars denote SE

**Fig. 5:**
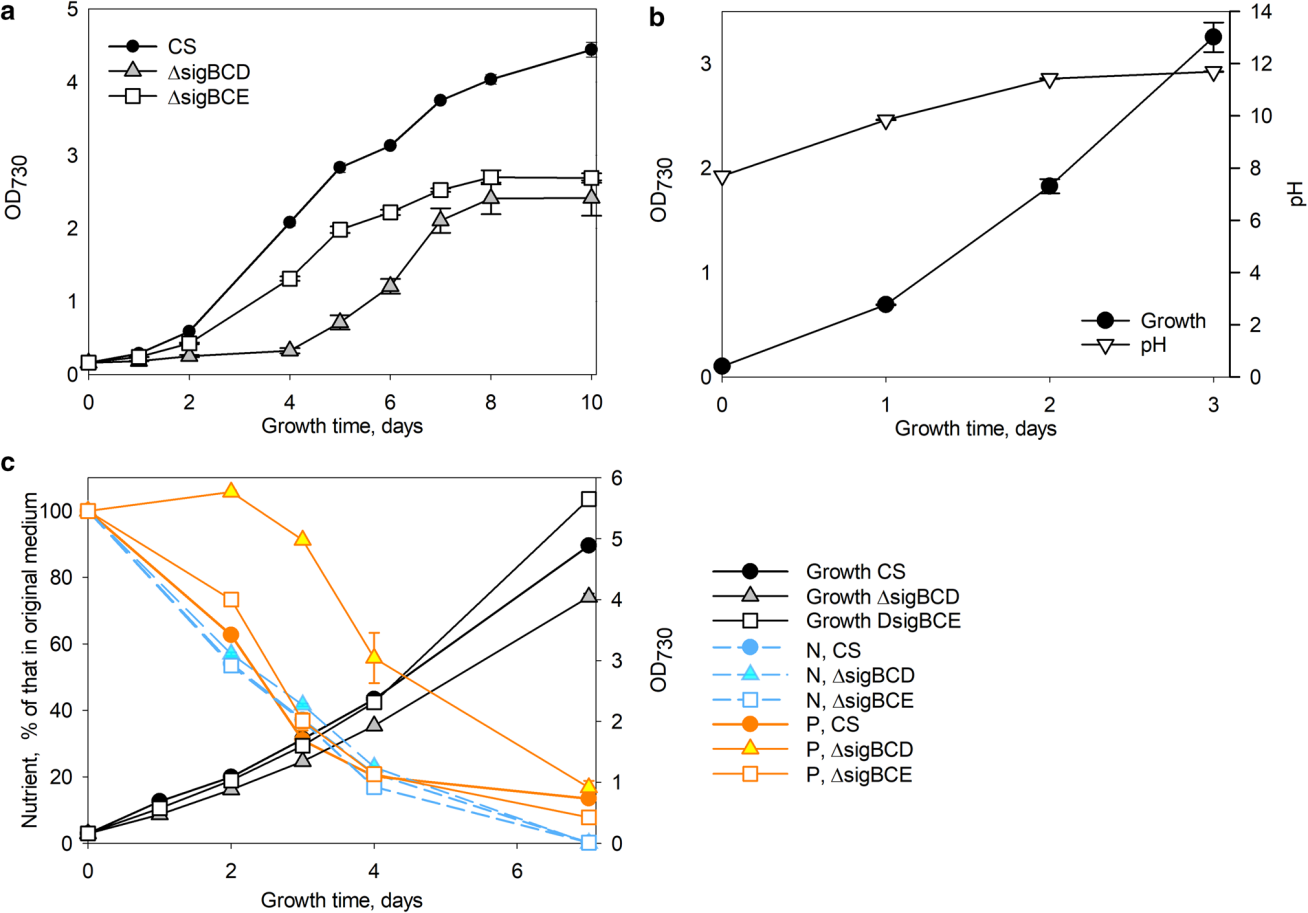
Effects of phosphate and pH on growth and nutrient removal. **a** The control, ΔsigBCD, and ΔsigBCE strains of *Synechocystis* were grown in KA2 wastewater supplemented with BG-11 level phosphate under constant illumination, PPFD 40 µmol m^−2^ s^−1^, at 32 °C in ambient air. **b** The control strain of *Synechocystis* was grown in BG-11 medium without buffering under constant illumination, PPFD 40 µmol m^−2^ s^−1^, at 32 °C in ambient air, and cell density and pH of growth medium were measured. **c** The control, ΔsigBCD, and ΔsigBCE strains of *Synechocystis* were grown in KA2 wastewater supplemented with BG-11 level phosphate and 20 mM Hepes, pH 7.5, under constant illumination, PPFD 40 µmol m^−2^ s^−1^, at 32 °C in ambient air. Three independent biological replicates were measured. The error bars denote SE

Phosphate transporter genes are highly upregulated at transcriptional level in normal phosphate-replete BG-11 medium in the ΔsigBCD strain; the data shown in Supplemental Table S1 have been collected from previously published DNA microarray results ( [29], Geo accessions GSE117478, GSE50060 and GSE69981). The initial phosphate concentration of the wastewater was 41–44% of that in standard BG-11 medium, which raises the question whether phosphate limitation is likely the cause for the observed early entry of cells to the stationary phase in KA2 wastewater. For this, the growth of the *Synechocystis* sp. PCC6803 strains in BG-11-5%P medium that contains only 8.75 µM phosphate was tested (Fig. 4). The control strain grew well in BG-11-5%P medium for the tested four days. All mutant strains grew as well as the control strain for the first 2 days in BG-11-5%P (Fig. 4), indicating that they all were able to harness phosphate even when phosphate concentration was low. The growth of ΔsigBCD and ΔsigBCE strains decelerated considerably in BG-11-5%P medium (Fig. 4). As the quadruple mutant ΔsigBCDE grew well in BG-11-5%P medium, the results indicate that low phosphate sensitivity is not due to missing σ factors but caused by a combined effect of missing σ factors and overdose of the remaining group 2 σ factor (Table 1). To directly test if the low phosphate content of KA2 was the reason for the early cessation of growth in KA2, phosphate was added to KA2 so that the final phosphate concentration of KA2 was the same as in the BG-11 medium. However, this addition did not improve the growth of ΔsigBCD or ΔsigBCE strains, and actually ΔsigBCD cells had a long lag phase before they started to grow (Fig. 5a), indicating that low phosphate was not the main reason for early cessation of the growth of the ΔsigBCD or ΔsigBCE strains in KA2.

Unlike in typical growth experiments, KA2 wastewater was not buffered with 20 mM Hepes, pH 7.5, as expensive buffers could not be used in real wastewater treatments. When cyanobacteria are grown in ambient air without buffering, the pH of growth medium increases rapidly (Fig. 5b). In ammonium containing wastewaters, high pH would lead to conversion of ammonium to toxic volatile ammonia. To test consequences of that, the growth and nutrient uptake of CS, ΔsigBCD, and ΔsigBCE cells in wastewater supplemented with phosphate and buffered with 20 mM Hepes to pH 7.5 were measured. The removal of ammonium from KA2 after buffering (Fig. 5c) was slower than without buffering (Fig. 2), indicating that without buffering, volatile ammonia was escaping. No differences were detected in ammonium removal between CS, ΔsigBCD, and ΔsigBCE cells (Fig. 5c). The ΔsigBCE strain first grew like CS in buffered KA2 and growth was even better than that of the CS in the end of the experiment (Fig. 5c). Rapid growth of ΔsigBCE cells in the end of the experiment was accompanied with more rapid removal of phosphate than that in the control strain (Fig. 5c). Contrary to the ΔsigBCE strain, ΔsigBCD cells grew more slowly than CS cells even after buffering KA2 medium and ΔsigBCD cells collected phosphate more slowly from buffered KA2 than the other strains (Fig. 5c).

### ΔsigBCE Grows Rapidly in High Light in a Laboratory-scale Bioreactor

**Fig. 6:**
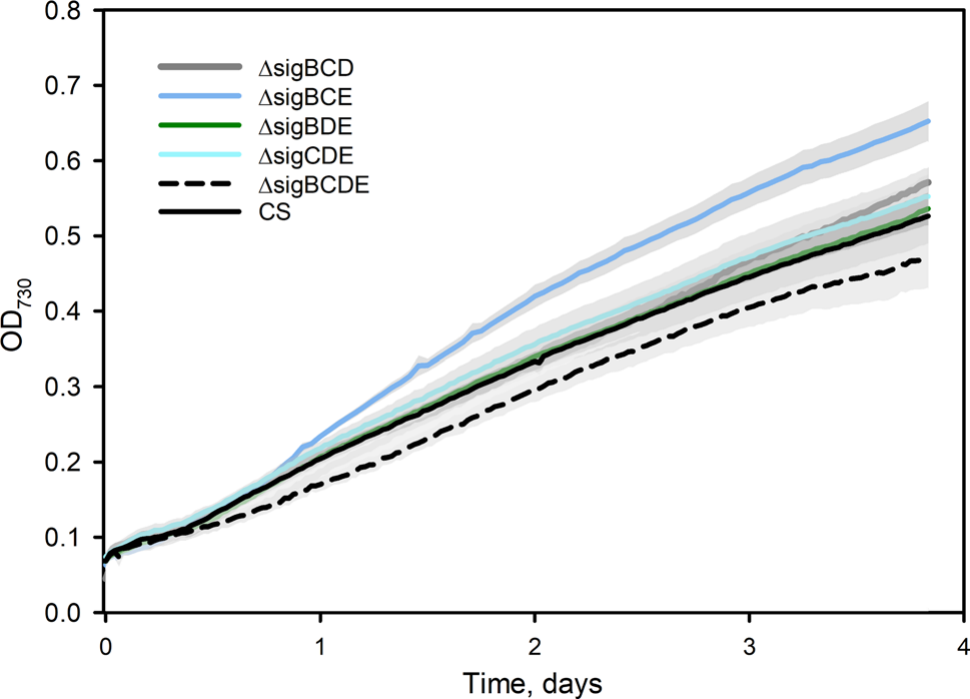
Growth of the control and σ factor inactivation strains in continuous bright light, PPFD 500 µmol m^−2^ s^−1^. Cells were grown in a Multicultivator. Lines represent average values from at least three independent measurements, and the grey areas show SE

An optimal cyanobacterial strain that would be utilized in wastewater treatment should tolerate not only nutrient imbalance or harmful substances but also non-optimal temperature and light conditions. The wastewater tests were done in standard laboratory condition. However, cyanobacteria utilized to treat wastewater can be expected to be exposed to brighter light than this laboratory standard, PPFD 40 µmol m^−2^ s^−1^. Next the ability of σ factor inactivation strains was tested to cope with high light in otherwise standard conditions. The growth in bright light was tested in a laboratory-scale bioreactor, "Multicultivator." The strain ΔsigBCE that contains only the SigD factor grew better than the control strain at PPFD 500 µmol m^−2^ s^−1^, and the slowest growth was measured for the ΔsigBCDE strain (Fig. 6). Thus, utilization of the ΔsigBCE strain might be useful if cells can be expected to experience high light.

## Discussion

The KA2 wastewater was found to be suitable for *Synechocystis* sp. PCC 6803 (Fig. 3). The group 2 σ factor less ΔsigBCDE strain is sensitive for many stress conditions, including oxidative stress (Table 1), and can therefore be considered as a stress-sensitive indicator strain. The growth of ΔsigBCDE in KA2 for few days indicates that KA2 does not induce immediate oxidative stress to *Synechocystis* sp. PCC 6803 cells (Fig. 3). The growth of cyanobacteria in KA2 wastewater removed almost all ammonium and phosphate within three days (Fig. 2). The removal of ammonium was slower if wastewater was buffered to 7.5 (Fig. 5c), indicating that part of the ammonium was released as ammonia in un-buffered wastewater and was not utilized by cyanobacteria. Cultivation of cyanobacteria in un-buffered medium under ambient air causes a rapid increase of pH (Fig. 5b), which facilitates conversion of ammonium to ammonia. Instead of using expensive buffers to keep the pH of the growth medium at the desired value, automatic addition of CO_2_ is used to counteract the growth-induced increase of pH in bioreactors [40]. It is shown that if growth chamber air is supplemented with 3% CO_2_, *Synechocystis* sp. PCC6803 cells grow very fast and the pH of un-buffered BG-11 remains 8.2 [9]. Thus, wastewaters containing ammonium might be possible to remediate with cyanobacteria in a pH-controlled process. For wastewaters containing nitrate as a nitrogen source, pH is not as critical. The results show that cyanobacteria are able to remove 90% of ammonium and phosphate within few days from wastewater, and they are also able to collect nitrate efficiently, taking into account that the nitrate concentration of BG-11 medium is 17.6 mM.

Cyanobacteria prefer ammonium as a nitrogen source [5, 41], and a regulatory network including the amounts of 2-oxoglutarate and ATP, and the regulatory proteins PII, PipX, and NtcA orchestrates nitrogen metabolism according to the C/N ratio, for a review see [8]. In nitrogen deficiency, abundant blue phycobiliproteins are degraded, followed by slow decrease of the yellow-green chl *a* and strong accumulation of protective carotenoids, which give the typical yellowish-brown color to cyanobacterial cultures in nitrogen limitation [32]. The wastewater cultures of the CS strain remained green throughout the whole experiment (Fig. 3) although growth was arrested earlier than in BG-11 medium, indicating that nitrogen deficiency might not be the main reason for early growth cessation.

*Synechocystis* sp. PCC cells consumed most of the phosphate of the standard BG-11 medium or KA2 wastewater within 3 days but grew well in BG-11-5%P for four days. Thus, phosphate deprivation is unlikely to be the main reason for the cessation of growth after six days in wastewater containing 8 times more phosphate than in BG-11-5%P. In cyanobacteria, phosphate taken from growth medium is deposited to an inner cellular pool and to an extracellular pool [42–44]. Cells first both take in phosphate and adsorb it with the extracellular matrix, and when the growth medium is depleted of phosphate, phosphate from the extracellular matrix is taken in [44]. Inside the cells, phosphate is stored as polyphosphate [17], and *Synechocystis* has been speculated to use multiple DNA copies as a phosphate storage as well, as at least the number of genome copies is dependent on the amount of phosphate [45].

The high expression of phosphate transporters in the ΔsigBCD strain in these standard growth conditions (Supplemental Table S1) and the early growth-arrest phenotype of ΔsigBCD in low-phosphate medium (Fig. 4) may indicate that ΔsigBCD has defects either in acquisition or in utilization of phosphate, and slower removal of phosphate by ΔsigBCD (Fig. 5c) indeed points to abnormalities in phosphate acquisition. The defects in phosphate acquisition/utilization are connected to the combined effects of missing SigB, SigC, and SigD factors and overdose of SigE factor (Table 1) as ΔsigBCDE strain missing all group 2 σ factors do not overexpress phosphate transporter genes (Supplemental Table S1) and grow better than ΔsigBCD in low-phosphate medium (Fig. 4). Expression of phosphate transporter genes is normal also in a SigE overexpression strain that contains all group 2 σ factors [33]. However, addition of extra phosphate did not improve growth of ΔsigBCD in KA2 (Fig. 5a). After buffering the KA2 wastewater (Fig. 5c), the long lag phase of ΔsigBCD strain shown in Fig. 5a disappeared. The ammonium concentrations of KA2 batch were 6.9 mM as shown in Fig. 5 and 5.9 mM as shown in Fig. 2, suggesting that toxic ammonia was formed in higher quantity as shown in Fig. 5a than that shown in Fig. 2a. Thus, it is possible that in addition to having a slowly functioning phosphate uptake system, the ΔsigBCD strain is also more sensitive to ammonia than CS. That would not be surprising, as both main oxidative stress responsive σ factors, SigB and SigD, are missing in ΔsigBCD [30].

Contrary to ΔsigBCD, the ΔsigBCE strain showed normal expression of phosphate transporter genes when grown in standard BG-11 medium. Unfortunately, transcriptome analysis of ΔsigBCE in standard conditions (Hakkila et al. 2019) does not give direct hints for possible mechanism(s), as the majority of up- or down-regulated genes produce proteins with unknown functions. Interestingly, pH adjustment of wastewater to 7.5 improved the growth of ΔsigBCE, and actually ΔsigBCE cells removed more phosphate and had higher cell density than the control strain in the end of the experiment (Fig. 5c). The ΔsigBCE strain that contains a high amount of the SigD factor grows better than the control strain also in high light (Fig. 6), singlet oxygen, or H_2_O_2_ stresses [30]. Thus, ΔsigBCE strain is potentially interesting for wastewater treatment as high light and reactive oxygen species tolerance might provide extra robustness required for wastewater treatment outside the strictly controlled laboratory conditions.

Cyanobacteria have already been tested for wastewater bioremediation, and the results are encouraging. A native *Synechocystis* sp. from Costa Rica was isolated and then nutrient removal was tested from synthetic medium containing same levels of phosphorus and nitrogen (95 µM phosphate and 3.9 mM ammonium) as Rio Azul and San José. *Synechocystis* sp. removed 70% of phosphate and 30% of ammonium within 5 days, however, green alga *Chlorella* was more efficient in removing both nutrients almost completely [46]. *Synechocystis* sp. was shown to remove phosphate in a recirculating aquaculture system [47], and other cyanobacterial species were shown to reduce the nitrogen content of dairy wastewater by circa 80% [48]. In accordance with these results, these results suggest that cyanobacteria might offer a potential solution for nitrogen and phosphorus removal from wastewaters.

## Electronic supplementary material

Below is the link to the electronic supplementary material.

Supplementary file1 (PDF 121 kb)
