# Supplementary material for: Testing the Potential of Regulatory Sigma Factor Mutants for Wastewater Purification or Bioreactor Run in High Light
Source: Curr Microbiol. 2020 Apr 7;77(8):1590–9. doi: 10.1007/s00284-020-01973-w (PMC7334282; doi:10.1007/s00284-020-01973-w)
Supplement: Supplementary file 1 — Supplementary file1 (PDF 121 kb) [file 284_2020_1973_MOESM1_ESM.pdf]

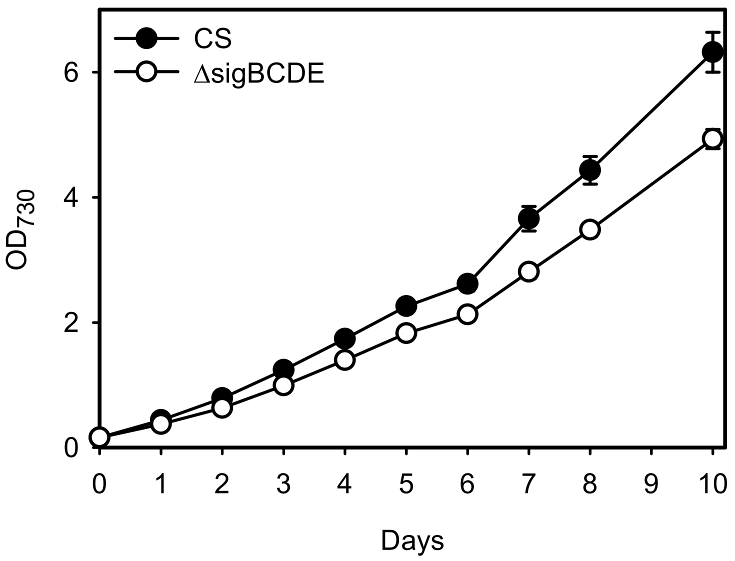


Fig S1. Growth of CS and ΔsigBCDE in standard growth conditions.

Supplemental Table S1. Expression of phosphate uptake systems in group 2 σ factor inactivation strains compared to the expression in the control strain. Results are shown as $log_2$ values of the fold change (FC). Transcriptome changes showing at least 4-fold difference between the mutant and control strain are shown in bold. DNA microarray results were collected from data published in Hakkila et al. (2019) Plant Cell Physiol 60:.436-447.

| | | | ΔsigBCD/CS | | ΔsigBCE/CS | | ΔsigBDE/CS | | ΔsigCDE/CS | | ΔsigBCDE/CS | |
|---|---|---|---|---|---|---|---|---|---|---|---|---|
| Code | Gene | Function | FC | p-value | FC | p-value | FC | p-value | FC | p-value | FC | p-value |
| sll0679 | sphX | SphX periplasmic phosphate binding protein | 3.68 | 0.0000 | -0.16 | 0.4728 | 0.31 | 0.1104 | -0.27 | 0.1532 | 0.45 | 0.0175 |
| sll0680 | pstS | Phosphate-binding periplasmic protein | 3.40 | 0.0000 | 0.37 | 0.2786 | 1.09 | 0.0035 | 0.13 | 0.7242 | 1.06 | 0.0010 |
| sll0681 | pstC | Phosphate transporter, permease PstC | 3.35 | 0.0000 | 0.28 | 0.1957 | 0.91 | 0.0012 | 0.18 | 0.2972 | 0.95 | 0.0004 |
| sll0682 | pstA | Phosphate transporter, permease PstA | 3.44 | 0.0000 | 0.27 | 0.2210 | 1.07 | 0.0004 | 0.08 | 0.7264 | 1.06 | 0.0001 |
| sll0683 | pstB | Phosphate transporter, ATP-binding protein PstB | 3.27 | 0.0000 | 0.17 | 0.5331 | 0.81 | 0.0031 | 0.06 | 0.8469 | 0.69 | 0.0029 |
| sll0684 | pstB | Phosphate transporter, ATP-binding protein PstB | 3.44 | 0.0000 | 0.31 | 0.1477 | 0.71 | 0.0021 | 0.31 | 0.0748 | 0.66 | 0.0010 |
| slr1247 | pstS | Phosphate-binding periplasmic protein | 6.78 | 0.0000 | -0.73 | 0.1045 | 0.66 | 0.0381 | 0.04 | 0.9668 | -0.20 | 0.4615 |
| slr1248 | pstC | Phosphate transporter, permease PstC | 6.62 | 0.0000 | -0.35 | 0.3306 | 1.02 | 0.0022 | -0.04 | 0.9526 | 0.87 | 0.0083 |
| slr1249 | pstA | Phosphate transporter, permease PstA | 5.47 | 0.0000 | -0.31 | 0.2122 | 0.48 | 0.0236 | 0.07 | 0.8164 | 0.48 | 0.0293 |
| slr1250 | pstB | Phosphate transporter, ATP-binding protein PstB | 4.36 | 0.0000 | 0.09 | 0.7902 | 0.45 | 0.0544 | 0.11 | 0.7459 | 0.41 | 0.0471 |
| sll0337 | sphS | SphS histidine kinase | -0.02 | 0.9252 | -0.36 | 0.1377 | 0.07 | 0.6943 | -0.13 | 0.5026 | 0.18 | 0.2150 |
| slr0081 | sphR | SphR response regulator | 0.97 | 0.0028 | 0.22 | 0.5146 | 0.55 | 0.0466 | 0.06 | 0.8827 | 0.74 | 0.0040 |
| slr0741 | sphU | SphU repressor protein | -0.87 | 0.0038 | -0.25 | 0.4262 | -0.10 | 0.7741 | 0.05 | 0.9018 | -0.46 | 0.0369 |
